# How Could Language Have Evolved?

**DOI:** 10.1371/journal.pbio.1001934

**Published:** 2014-08-26

**Authors:** Johan J. Bolhuis, Ian Tattersall, Noam Chomsky, Robert C. Berwick

**Affiliations:** 1 Cognitive Neurobiology and Helmholtz Institute, Departments of Psychology and Biology, Utrecht University, Utrecht, The Netherlands; 2 Department of Zoology and Sidney Sussex College, University of Cambridge, Cambridge, United Kingdom; 3 Division of Anthropology, American Museum of Natural History, New York, New York, United States of America; 4 Department of Linguistics and Philosophy, MIT, Cambridge, Massachusetts, United States of America; 5 Department of Electrical Engineering & Computer Science and Brain and Cognitive Sciences, MIT, Cambridge, Massachusetts, United States of America

## Abstract

The evolution of the faculty of language largely remains an enigma. In this essay, we ask why. Language's evolutionary analysis is complicated because it has no equivalent in any nonhuman species. There is also no consensus regarding the essential nature of the language “phenotype.” According to the “Strong Minimalist Thesis,” the key distinguishing feature of language (and what evolutionary theory must explain) is hierarchical syntactic structure. The faculty of language is likely to have emerged quite recently in evolutionary terms, some 70,000–100,000 years ago, and does not seem to have undergone modification since then, though individual languages do of course change over time, operating within this basic framework. The recent emergence of language and its stability are both consistent with the Strong Minimalist Thesis, which has at its core a single repeatable operation that takes exactly two syntactic elements *a* and *b* and assembles them to form the set {a, b}.

## Abstract 2

How could language have evolved? What is the key innovation underlying the evolution of human language? This Essay argues that the ability to “merge” two syntactic elements uniquely explains the recentness and stability of language. [SK to check before publishing on the homepage] CM 16/7

It is uncontroversial that language has evolved, just like any other trait of living organisms. That is, once—not so long ago in evolutionary terms—there was no language at all, and now there is, at least in *Homo sapiens*. There is considerably less agreement as to how language evolved. There are a number of reasons for this lack of agreement. First, “language” is not always clearly defined, and this lack of clarity regarding the language phenotype leads to a corresponding lack of clarity regarding its evolutionary origins. Second, there is often confusion as to the nature of the evolutionary process and what it can tell us about the mechanisms of language. Here we argue that the basic principle that underlies language's hierarchical syntactic structure is consistent with a relatively recent evolutionary emergence.

## Conceptualizations of Language

The language faculty is often equated with “communication”—a trait that is shared by all animal species and possibly also by plants. In our view, for the purposes of scientific understanding, language should be understood as a particular computational cognitive system, implemented neurally, that cannot be equated with an excessively expansive notion of “language as communication” [1]. Externalized language may be used for communication, but that particular function is largely irrelevant in this context. Thus, the origin of the language faculty does not generally seem to be informed by considerations of the evolution of communication. This viewpoint does not preclude the possibility that communicative considerations can play a role in accounting for the maintenance of language once it has appeared or for the historical language change that has clearly occurred within the human species, with all individuals sharing a common language faculty, as some mathematical models indicate [1]–[3]. A similar misconception is that language is coextensive with speech and that the evolution of vocalization or auditory-vocal learning can therefore inform us about the evolution of language (Box 1) [1],[4]. However, speech and speech perception, while functioning as possible external interfaces for the language system, are not identical to it. An alternative externalization of language is in the visual domain, as sign language [1]; even haptic externalization by touch seems possible in deaf and blind individuals [5]. Thus, while the evolution of auditory-vocal learning may be relevant for the evolution of speech, it is not for the language faculty per se. We maintain that language is a computational cognitive mechanism that has hierarchical syntactic structure at its core [1], as outlined in the next section.

**Box 1. Comparative Linguistics: Not Much to Compare**

A major stumbling block for the comparative analysis of language evolution is that, so far, there is no evidence for human-like language syntax in any nonhuman species [4],[41],[42]. There is no a priori reason why a version of such a combinatorial computational system could not have evolved in nonhuman animals, either through common descent (e.g., apes) or convergent evolution (e.g., songbirds) [1],[18]. Although the auditory-vocal domain is just one possible external interface for language (with signing being another), it could be argued that the strongest animal candidates for human-like syntax are songbirds and parrots [1],[41],[42]. Not only do they have a similar brain organization underlying auditory-vocal behavior [4],[43],[44], they also exhibit vocal imitation learning that proceeds in a very similar way to speech acquisition in human infants [4],[41],[42]. This ability is absent in our closest relatives, the great apes [1],[4]. In addition, like human spoken language, birdsong involves patterned vocalizations that can be quite complex, with a set of rules that govern variable song element sequences known as “phonological syntax” [1],[4],[41],[42],[45]. Contrary to recent suggestions [46],[47], to date there is no evidence to suggest that birdsong patterns exhibit the hierarchical syntactic structure that characterizes human language [41],[48],[49] or any mapping to a level forming a language of thought as in humans. Avian vocal-learning species such as parrots are able to synchronize their behavior to variable rhythmic patterns [50]. Such rhythmic abilities may be involved in human prosodic processing, which is known to be an important factor in language acquisition [51].

## The Faculty of Language According to the “Strong Minimalist Thesis”

In the last few years, certain linguistic theories have arrived at a much more narrowly defined and precise phenotype characterizing human language syntax. In place of a complex rule system or accounts grounded on general notions of “culture” or “communication,” it appears that human language syntax can be defined in an extremely simple way that makes conventional evolutionary explanations much simpler. In this view, human language syntax can be characterized via a single operation that takes exactly two (syntactic) elements *a* and *b* and puts them together to form the set {a, b}. We call this basic operation “*merge*” [1]. The “Strong Minimalist Thesis” (SMT) [6] holds that *merge* along with a general cognitive requirement for computationally minimal or efficient search suffices to account for much of human language syntax. The SMT also requires two mappings: one to an internal conceptual interface for thought and a second to a sensory-motor interface that externalizes language as speech, sign, or other modality [1]. The basic operation itself is simple. Given *merge*, two items such as *the* and *apples* are assembled as the set {*the, apples*}. Crucially, *merge* can apply to the results of its own output so that a further application of *merge* to *ate* and {*the, apples*} yields the set {*ate*, {*the, apples*}}, in this way deriving the full range of characteristic hierarchical structure that distinguishes human language from all other known nonhuman cognitive systems.

**Figure 1:**
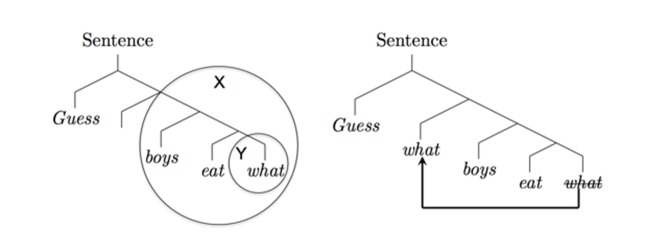
The binary operation of *merge* (X,Y) when Y is a subset of X leads to the ubiquitous phenomenon of “displacement” in human language, as in *Guess what boys eat*. Left: The circled structure Y, corresponding to *what*, the object of the verb *eat*, is a subset of the circled structure X, corresponding to *boys eat what*. Right: The free application of *merge* to X, Y in this case automatically leads to *what* occupying two syntactic positions, as required for proper semantic interpretation. The original *what* remains as the object of the verb so that it can serve as an argument to this predicate, and a copy of *what*, “displaced,” is now in the position of a quantificational operator so that the form can be interpreted as “for what *x*, boys eat *x*.” Typically, only the higher *what* is actually pronounced, as indicated by the line drawn through the lower *what*.

As the text below and Figure 1 shows, *merge* also accounts for the characteristic appearance of displacement in human language—the apparent “movement” of phrases from one position to another. Displacement is not found in artificially constructed languages like computer programming languages and raises difficulties for parsing as well as communication. On the SMT account, however, displacement arises naturally and is to be expected, rather than exceptional, as seems true in every human language that has been examined carefully. Furthermore, hierarchical language structure is demonstrably present in humans, as shown, for instance, by online brain imaging experiments [7], but absent in nonhuman species, e.g., chimpanzees taught sign language demonstrably lack this combinatorial ability [8]. Thus, before the appearance of *merge*, there was no faculty of language as such, because this requires *merge* along with the conceptual atoms of the lexicon. Absent this, there is no way to arrive at the essentially infinite number of syntactic language structures, e.g., “the brown cow,” “a black cat behind the mat” [9]–[11], etc. This view leaves room for the possibility that some conceptual atoms were present antecedent to *merge* itself, though at present this remains entirely speculative. Even if true, there seems to be no evidence for an antecedent combinatorial and hierarchical syntax. Furthermore, *merge* itself is uniform in the contemporary human population as well as in the historical record, in contrast to human group differences such as the adult ability to digest lactose or skin pigmentation [12]. There is no doubt that a normal child from England raised in northern Alaska would readily learn Eskimo-Aleut, or vice versa; there have been no confirmed group differences in the ability of children to learn their first language, despite one or two marginal, indirect, and as yet unsubstantiated correlative indications [13]. This uniformity and stability points to the absence of major evolutionary change since the emergence of the language faculty. Taken together, these facts provide good evidence that *merge* was indeed the key evolutionary innovation for the language faculty.

It is sometimes suggested that external motor sequences are “hierarchical” in this sense and so provide an antecedent platform for language [14]. However, as has been argued [15], motor sequences resemble more the “sequence of letters in the alphabet than the sequences of words in a sentence” ([15], p. 221). (For expository purposes, we omit here several technical linguistic details about the labelling of these words; see [16].) Along with the conceptual atoms of the lexicon, the SMT holds that *merge*, plus the internal interface mappings to the conceptual system, yields what has been called the “language of thought” [17].

More narrowly, the SMT also suffices to automatically derive some of the most central properties of human language syntax. For example, one of the most distinctive properties of human language syntax is that of “displacement,” along with what is sometimes called “duality of semantic patterning.” For example, in the sentence “(Guess) what boys eat,” “what” takes on a dual role and is interpreted in two places: first, as a question “operator” at the front of the sentence, where it is pronounced; and second, as a variable that serves as the argument of the verb *eat*, the thing eaten, where it is not pronounced (Figure 1). (There are marginal exceptions to the nonpronunciation of the second “what” that, when analyzed carefully, support the picture outlined here.) Given the free application of *merge*, we expect human languages to exhibit this phenomenon of displacement without any further stipulation. This is simply because operating freely, without any further constraints, *merge* derives this possibility. In our example “(Guess) what boys eat,” we assume that successive applications of *merge* as in our earlier example will first derive {*boys*, {*eat, what*}}—analogous to {*boys*, {*eat, apples*}}. Now we note that one can simply apply *merge* to the two syntactic objects {*boys*,{*eat, what*}} and {*what*}, in which {*what*} is a subcomponent (a subset) of the first syntactic object rather than some external set. This yields something like {*what*, {*boys*, {*eat, what*}}}, in this way marking out the two required operator and variable positions for *what*.

## The Nature of Evolution

Evolutionary analysis might be brought to bear on language in two different ways. First, evolutionary considerations could be used to explain the mechanisms of human language. For instance, principles derived from studying the evolution of communication might be used to predict, or even explain, the structural organization of language. This approach is fraught with difficulties. Questions of evolution or function are fundamentally different from those relating to mechanism, so evolution can never “explain” mechanisms [18]. For a start, the evolution of a particular trait may have proceeded in different ways, such as via common descent, convergence, or exaptation, and it is not easy to establish which of these possibilities (or combination of them) is relevant [18],[19]. More importantly, evolution by natural selection is not a causal factor of either cognitive or neural mechanisms [18]. Natural selection can be seen as one causal factor for the historical process of evolutionary change, but that is merely stating the essence of the theory of evolution. As we have argued, communication cannot be equated with language, so its evolution cannot inform the mechanisms of language syntax. However, evolutionary considerations—in particular, reconstructing the evolutionary history of relevant traits—might provide clues or hypotheses as to mechanisms, even though such hypotheses have frequently been shown to be false or misleading [18]. One such evolutionary clue is that, contrary to received wisdom, recent analyses suggest that significant genetic change may occur in human populations over the course of a few hundred years [19]. Such rapid change could also have occurred in the case of language, as we will argue below. In addition, as detailed in the next section, paleoanthropological evidence suggests that the appearance of symbolic thought, our most accurate proxy for language, was a recent evolutionary event. For instance, the first evidence of putatively symbolic artifacts dates back to only around 100,000 years ago, significantly after the appearance on the planet of anatomically distinctive *Homo sapiens* around 200,000 years ago [20],[21],

The second, more traditional way of applying evolutionary analysis to language is to attempt to reconstruct its evolutionary history. Here, too, we are confronted with major explanatory obstacles. For starters, language appears to be unique to the species *H. sapiens*. That eliminates one of the cornerstones of evolutionary analysis, the comparative method, which generally relies on features that are shared by virtue of common descent (Box 1) [1],[4],[18]. Alternatively, analysis can appeal to convergent evolution, in which similar features, such as birds' wings and bats' wings, arise independently to “solve” functionally analogous problems. Both situations help constrain and guide evolutionary explanation. Lacking both, as in the case of language, makes the explanatory search more difficult. In addition, evolutionary analysis of language is often plagued by popular, naïve, or antiquated conceptions of how evolution proceeds [19],[22]. That is, evolution is often seen as necessarily a slow, incremental process that unfolds gradually over the eons. Such a view of evolutionary change is not consistent with current evidence and our current understanding, in which evolutionary change can be swift, operating within just a few generations, whether it be in relation to finches' beaks on the Galapagos, insect resistance to pesticides following WWII, or human development of lactose tolerance within dairy culture societies, to name a few cases out of many [19],[22]–[24].

## Paleoanthropology

**Figure 2:**
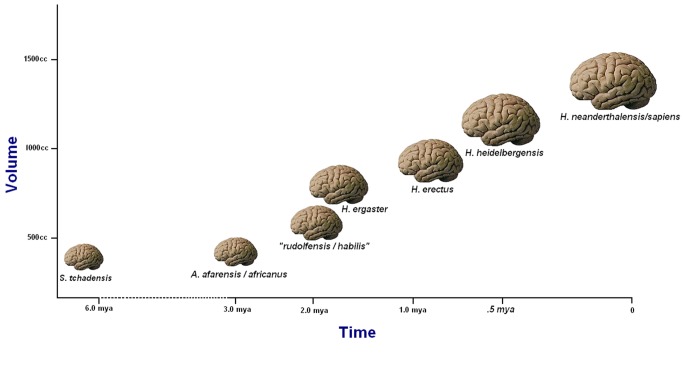
A crude plot of average hominid brain sizes over time. Although after an initial flatlining this plot appears to show consistent enlargement of hominid brains over the last 2 million years, it is essential to note that these brain volumes are averaged across a number of independent lineages within the genus *Homo* and likely represent the preferential success of larger-brained species. From [20]. *Image credit: Gisselle Garcia, artist (brain images)*.

Language leaves no direct imprint in the fossil record, and the signals imparted by putative morphological proxies are highly mixed. Most of these involve speech production and detection, neither of which by itself is sufficient for inferring language (see Box 2). After all, while the anatomical potential to produce the frequencies used in modern speech may be necessary for the expression of language, it provides no proof that language itself was actually employed. What is more, it is not even necessary for language, as the visual and haptic externalization routes make clear. Moreover, even granting that speech is a requirement for language, it has been argued convincingly [25],[26] that equal proportions of the horizontal and vertical portions of the vocal tract are necessary for producing speech. This conformation is uniquely seen in our own species *Homo sapiens*. In a similar vein, the aural ability of nonhuman primates like chimpanzees or extinct hominid species such as *H. neanderthalensis* to perceive the sound frequencies associated with speech [26],[27] says nothing about the ability of these relatives to understand or produce language. Finally, neither the absolute size of the brain nor its external morphology as seen in endocasts has been shown to be relevant to the possession of language in an extinct hominid (Figure 2) [28]. Recent research has determined that Neanderthals possessed the modern version of the FOXP2 gene [29], malfunctions in which produce speech deficits in modern people [4],[30]. However, FOXP2 cannot be regarded as “the” gene “for” language, since it is only one of many that have to be functioning properly to permit its normal expression.

**Box 2. The Infamous Hyoid Bone**

A putative relationship between basicranial flexion, laryngeal descent, and the ability to produce sounds essential to speech was suggested [52] before any fossil hyoid bones, the sole hard-tissue components of the laryngeal apparatus, were known. It was speculated that fossil hyoids would indicate when speech, and by extension language, originated. A Neanderthal hyoid from Kebara in Israel eventually proved very similar to its *H. sapiens* homologue, prompting the declaration that speech capacity was fully developed in adult *H. neanderthalensis* [53]. This was soon contested on the grounds that the morphology of the hyoid is both subsidiary [25] and unrelated [26] to its still-controversial [36] position in the neck. A recent study [54] focuses on the biomechanics, internal architecture, and function of the Kebara fossil. The authors conclude that their results “add support for the proposition that the Kebara 2 Neanderthal engaged in speech” ([54], p. 6). However, they wisely add that the issue of Neanderthal language will be fully resolved only on the basis of fuller comparative material. While the peripheral ability to produce speech is undoubtedly a necessary condition for the expression of vocally externalized language, it is not a sufficient one, and hyoid morphology, like most other lines of evidence, is evidently no silver bullet for determining when human language originated.

In terms of historically calibrated records, this leaves us only with archaeology, the archive of ancient human behaviors—although we have once again to seek indirect proxies for language. To the extent that language is interdependent with symbolic thought [20], the best proxies in this domain are objects that are explicitly symbolic in nature. Opinions have varied greatly as to what constitutes a symbolic object, but if one excludes stone and other Paleolithic implements from this category on the fairly firm grounds that they are pragmatic and that the techniques for making them can be passed along strictly by imitation [31], we are left with objects from the African Middle Stone Age (MSA) such as pierced shell beads from various ∼100,000-year-old sites (e.g., [32]) and the ∼80,000-year-old geometrically engraved plaques from South Africa's Blombos Cave [33] as the earliest undisputed symbolic objects. Such objects began to be made only substantially after the appearance, around 200,000 years ago, of anatomically recognizable *H. sapiens*, also in Africa [34]. To be sure, this inference from the symbolic record, like much else in paleontology, rests on evidence that is necessarily quite indirect. Nevertheless, the conclusion lines up with what is known from genomics.

Our species was born in a technologically archaic context [35], and significantly, the tempo of change only began picking up after the point at which symbolic objects appeared. Evidently, a new potential for symbolic thought was born with our anatomically distinctive species, but it was only expressed after a necessary cultural stimulus had exerted itself. This stimulus was most plausibly the appearance of language in members of a species that demonstrably already possessed the peripheral vocal apparatus required to externalize it [20],[22]. Then, within a remarkably short space of time, art was invented, cities were born, and people had reached the moon. By this reckoning, the language faculty is an extremely recent acquisition in our lineage, and it was acquired not in the context of slow, gradual modification of preexisting systems under natural selection but in a single, rapid, emergent event that built upon those prior systems but was not predicted by them. It may be relevant to note that the anatomical ability to express language through speech was acquired at a considerable cost, namely the not-insignificant risk of adults choking to death [25],[36], as simultaneous breathing and swallowing became impossible with the descent of the larynx. However, since this conformation was already in place before language had demonstrably been acquired (see Box 2), the ability to express language cannot by itself have been the countervailing advantage. Finally, there has been no detectable evolution of the language faculty since it emerged, with no known group differences. This is another signature of relatively recent and rapid origin. For reasons like these, the relatively sudden origin of language poses difficulties that may be called “Darwin's problem.”

## The Minimalist Account of Language—Progress towards Resolving “Darwin's Problem”

The Strong Minimalist Thesis (SMT) [6], as discussed above, greatly eases the explanatory burden for evolutionary analysis, since virtually all of the antecedent “machinery” for language is presumed to have been present long before the human species appeared. For instance, it appears that the ability to perceive “distinctive features” such as the difference between the sound *b*, as in *bat*, as opposed to *p*, as in *pat*, might be present in the mammalian lineage generally [37],[38]. The same holds for audition. Both comprise part of the externalization system for language. Furthermore, the general constraint of efficient computation would also seem plausibly antecedent in the cognitive computation of ancestral species. The only thing lacking for language would be *merge*, some specific way to externalize the internal computations and, importantly, the “atomic conceptual elements” that we have identified with words. Without *merge*, there would be no way to assemble the arbitrarily large, hierarchically structured objects with their specific interpretations in the language of thought that distinguish human language from other animal cognitive systems—just as Darwin insisted: “A complex train of thought can be no more carried out without the use of words, whether spoken or silent, than a long calculation without the use of figures or algebra” ([39], p. 88). With *merge*, however, the basic properties of human language emerge. Evolutionary analysis can thus be focused on this quite narrowly defined phenotypic property, *merge* itself, as the chief bridge between the ancestral and modern states for language. Since this change is relatively minor, it accords with what we know about the apparent rapidity of language's emergence.

## Conclusions

The Strong Minimalist Thesis that we have sketched here is consistent with a recent and rapid evolutionary emergence of language. Although this thesis is far from being established and contains many open questions, it offers an account that is compatible with the known empirical evolutionary evidence. Such an account also aligns with what we currently know about the relatively few genomic differences between our species and other ancestral *Homo* species—e.g., only about 100 coding gene differences between *Homo sapiens* and *H. neanderthalensis*, the majority of them in nonlanguage areas such as the olfactory and immune systems [40]. Furthermore, as far as we can tell from direct historical evidence, the capacity that emerged, namely the ability of any child to learn any human language, has remained frozen for 10,000 years or more. To be sure, such observations must be interpreted with great care and can remain only suggestive as long as we lack the knowledge to even crudely connect genomic changes to the relevant phenotypes. Even given these caveats, it appears that there has simply not been enough time for large-scale evolutionary changes, as indicated by the SMT. Clearly, such a novel computational system could have led to a large competitive advantage among the early *H. sapiens* who possessed it, particularly when linked to possibly preexisting perceptual and motor mechanisms.
